# Pulmonary perfusion with oxygenated blood or custodiol HTK solution during cardiac surgery for postoperative pulmonary function in COPD patients: a trial protocol for the randomized, clinical, parallel group, assessor and data analyst blinded Pulmonary Protection Trial

**DOI:** 10.1186/1745-6215-14-30

**Published:** 2013-01-31

**Authors:** Katrine B Buggeskov, Jørn Wetterslev, Niels H Secher, Lars W Andersen, Thomas Jonassen, Daniel A Steinbrüchel

**Affiliations:** 1 Department of Thoracic Anaesthesiology, Copenhagen University Hospital, Rigshospitalet, The Heart Centre dept. 4142, Blegdamsvej 9, 2100, Copenhagen, Denmark; 2 Department of Cardiothoracic Surgery, Rigshospitalet, The Heart Centre dept. 2.15.2, Blegdamsvej 9, 2100, Copenhagen, Denmark; 3 Department of Anaesthesiology, Rigshospitalet, Blegdamsvej 9, 2100, Copenhagen, Denmark; 4 Department of Cardiothoracic Anaesthesiology, Rigshospitalet, The Heart Centre dept. 4142, The University of Copenhagen, Blegdamsvej 3, 2200, Copenhagen, Denmark; 5 Department of Biomedical Sciences, Blegdamsvej 9, 2100, Copenhagen, Denmark; 6 The Copenhagen Trial Unit, Rigshospitalet, Centre for Clinical Intervention Research, Blegdamsvej 9, 2100, Copenhagen, Denmark

**Keywords:** Cardiopulmonary bypass, Inflammation, Systemic inflammatory response syndrome, Pulmonary function, Pulmonary perfusion, Pulmoplegia, Transcatheter aortic-valve implantation

## Abstract

**Background:**

Five to thirty percent of patients undergoing cardiac surgery present with chronic obstructive pulmonary disease (COPD) and have a 2- to 10-fold higher 30-day mortality risk. Cardiopulmonary bypass (CPB) creates a whole body systemic inflammatory response syndrome (SIRS) that could impair pulmonary function. Impaired pulmonary function can, however, be attenuated by pulmonary perfusion with oxygenated blood or custodiol HTK (histidine-tryptophan-ketoglutarate) solution.

**Methods/Design:**

The Pulmonary Protection Trial (PP-Trial) randomizes 90 patients undergoing CPB-dependent cardiac surgery to evaluate whether pulmonary perfusion with oxygenated blood or custodiol HTK solution reduces postoperative pulmonary dysfunction in COPD patients. Further, we aim for a non-randomized evaluation of postoperative pulmonary function after transcatheter aortic-valve implantation (TAVI). The primary outcome measure is the oxygenation index measured from anesthesia induction to the end of surgery and until 24 hours after anesthesia induction for a total of six evaluations.

**Discussion:**

Patients with COPD may be impaired by hypoxemia and SIRS. Thus, prolonged recovery and even postoperative complications and death may be reflected by the degree of hypoxemia and SIRS. The limited sample size does not aim for confirmatory conclusions on mortality, cardiovascular complications or risk of pneumonia and sepsis, but the PP-Trial is considered an important feasibility trial paving the road for a multicenter confirmatory trial.

**Trial registration:**

ClinicalTrials.gov: NCT01614951.

## Background

Pulmonary dysfunction is a complication of cardiac surgery with cardiopulmonary bypass (CPB) and yet a preoperative pulmonary function test (PFT) is not performed routinely although lung function appears to be an independent predictor of both morbidity and mortality [1]. Five to thirty percent of patients undergoing cardiac surgery present with chronic obstructive pulmonary disease (COPD), and patients with moderate to severe COPD, including a fifty percent reduction in diffusion capacity, demonstrate a 2- to 10-fold higher 30-day mortality risk [1-3].

Depending on the preoperative lung function, pulmonary dysfunction varies from only a short need of mechanical ventilation to fever, productive cough, pulmonary edema, respiratory failure, and consequent prolonged ICU - length of stay (ICU-LOS) and, in the most severe cases, adult respiratory distress syndrome (ARDS) with a mortality rate of approximately 50% [4-6].

The etiology of pulmonary dysfunction after CPB-dependent cardiac surgery is multifactorial [4,5,7-9]. However, contact of blood with artificial surfaces creates a whole body systemic inflammatory response syndrome (SIRS), lung ischemia and reperfusion injury with increases in immunologic mediators and CPB-induced non-physiologic laminar perfusion, including release of endotoxins from the splanchnicus area, are considered reasons for postoperative pulmonary dysfunction [4,5,7-9].

In clinical trials for patients with a preoperative normal lung function, the reduction in PF ratio (PF = P_a_O_2_/F_i_O_2_) and increase in immunologic mediators are attenuated by pulmonary perfusion with oxygenated blood [10,11] or custodiol HTK (histidine-tryptophan-ketoglutarate) solution [12].

The custodiol HTK solution is similar to the chemical composition of extracellular fluid, including a low potassium concentration, and is used for flushing donor liver, kidney, heart, lung, and pancreas prior to organ harvesting [13].

Alternatively to CPB- dependent aortic valve replacement (AVR), transcatheter aortic-valve implantation (TAVI) represents a procedure during which the aortic valve is replaced via inguinal access without the use of CPB. Procedural stress, including the inflammatory response is, therefore, expected to be reduced. Whether the TAVI procedure preserves lung function remains, however, unknown.

This trial evaluates whether pulmonary perfusion with oxygenated blood or custodiol HTK solution reduces postoperative pulmonary dysfunction after CPB-dependent cardiac surgery in patients with COPD. Further, we aim for a non-randomized evaluation of postoperative pulmonary function after TAVI in COPD patients compared to COPD patients undergoing CPB-dependent cardiac surgery, with or without pulmonary perfusion.

### Aims

This trial assesses 1) the effect of pulmonary perfusion with oxygenated blood or custodiol HTK solution during CPB on postoperative arterial oxygenation in COPD patients undergoing cardiac surgery, and 2) postoperative arterial oxygenation after TAVI compared to CPB-dependent cardiac surgery, with or without pulmonary perfusion in COPD patients.

## Methods/Design

The trial is designed as an open, assessor and data analyst blinded, randomized clinical trial on patients with COPD undergoing cardiac surgery. The patients will be randomized 1:1:1 to pulmonary perfusion with oxygenated blood or custodiol HTK solution during CPB, while patients who are not provided with selective pulmonary perfusion during CPB and those undergoing TAVI serve as control groups. The randomization procedure will be stratified according to the severity of COPD since we expect a relation between the responses to the interventions and the severity of COPD.

Patients having a TAVI are currently randomized to either CPB-dependent aortic valve replacement or TAVI (ClinicalTrials.gov: NCT01057173) and therefore will patients undergoing TAVI not be randomized for the Pulmonary Protection Trial (PP-Trial) because they do not receive any intervention. Patients undergoing TAVI serve, however, as controls for the intervention groups in the PP-Trial and will be enrolled when approximately half of the patients are randomized to pulmonary perfusion with oxygenated blood or custodiol HTK solution, or control. The distribution of the severity of COPD in the TAVI patients will be attempted to be identical with the distribution in the randomized groups.

### Inclusion criteria

1. The patients included in the trial undergo either

• Coronary artery bypass graft (CABG)

• AVR

• CABG + AVR

• TAVI

2. The preoperative PFT indicates COPD defined as FEV_1_/FVC ≤70% [14].

3. Informed consent is obtained from each patient.

### Exclusion criteria

• Age <18 years.

• Previous surgery of the heart or lungs.

• Previous thoracic exposure to radiation.

• Preoperative heart failure (ejection fraction <20%).

• A preoperative heart rate >100 bpm and/or a systolic blood pressure <100 mmHg.

• Tracheal intubation before surgery.

• Treatment with antibiotics for pneumonia.

• Hemodialysis.

• Patients who are pregnant or nursing.

### Randomization

**Figure 1:**
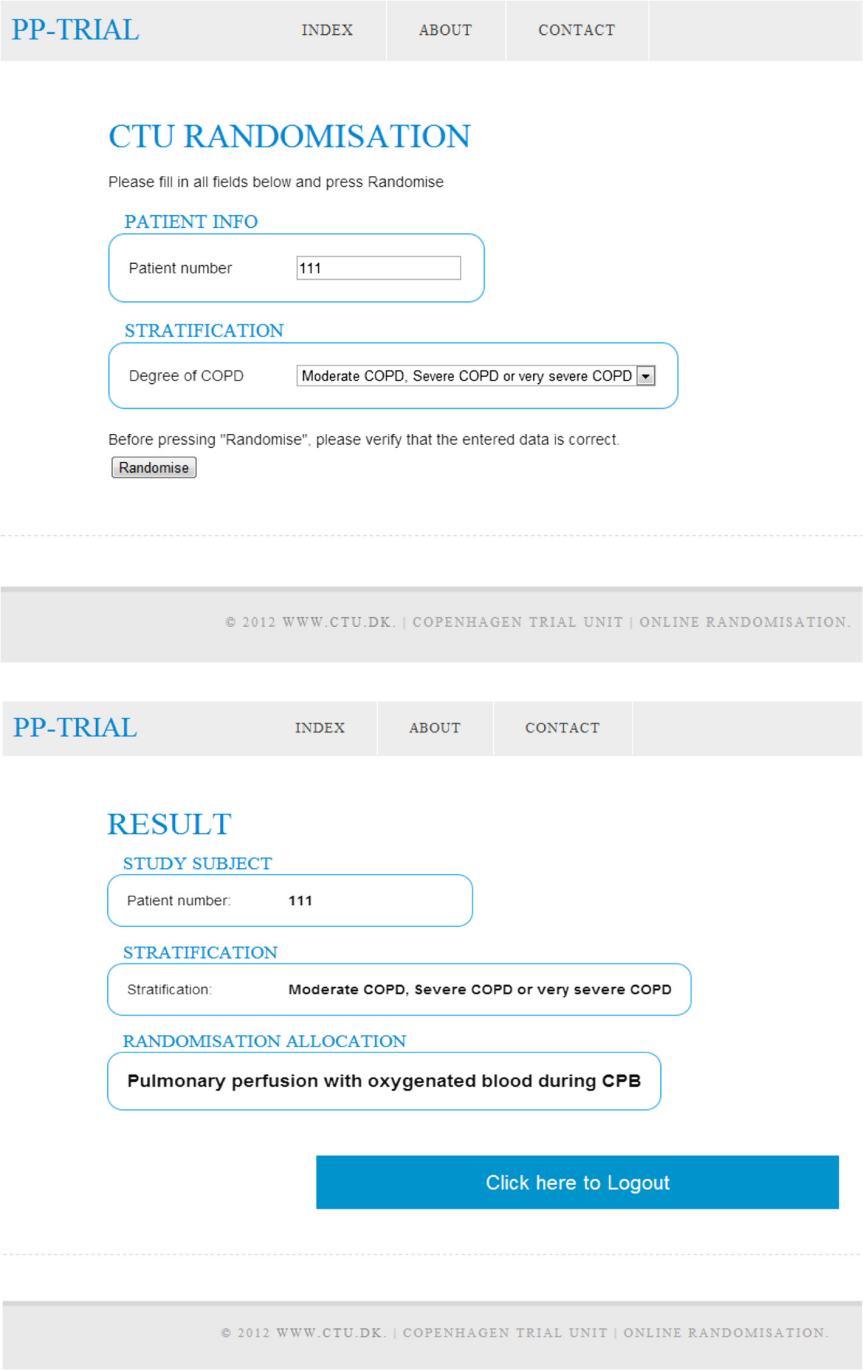
**Randomization.** Step 1 and 2 in randomization of a patient to intervention with, in this example, pulmonary perfusion with oxygenated blood during CPB generated by a web-based system following successful screening and stratification of a patient (all inclusion criteria fulfilled, no exclusion criteria fulfilled, informed consent obtained). CPB, cardiopulmonary bypass.

Staff at the trial site have 24-hour access to an internet-based randomization system developed by The Copenhagen Trial Unit (CTU) to allow for concealed allocation and intervention with pulmonary perfusion with blood or pulmoplegia versus no such intervention [Figure 1].

**Figure 2:**
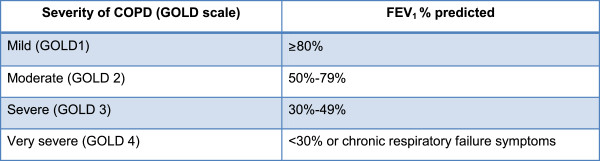
**Classification of COPD.** The limit is set out in international recommendations [15,16]. COPD, chronic obstructive pulmonary disease.

The allocation sequence is computer-generated with a variable block size that is unknown to the investigators. Randomization of the patients is stratified into two groups according to preoperative lung function: 1) mild COPD and 2) moderate, severe or very severe COPD [Figure 2]. We expect a 50/50 distribution of the patients into the two groups since there are only a few or no patients operated who present with severe or very severe COPD.

Each patient will be provided with a unique patient- and randomization number.

### Primary outcome measure

The primary outcome measure will be the oxygenation index (OI) that is measured from anesthesia induction, to the end of surgery and until 24 hours after induction for a total of six evaluations.

### Secondary outcome measures

• Oral tracheal intubation after surgery (hours).

• ICU-LOS after surgery (hours).

• Number of pneumonia and ventilator-associated pneumonia (VAP) 90 days after randomization.

• Bleeding and number of blood transfusion products administered during surgery and within the first 24 hours after surgery.

• Number of reoperations due to hemorrhage.

• Decrease in thoracic electrical impedance in reflection of accumulation of extra-vascular lung water (EVLW) during surgery and within the first 24 hours thereafter.

• Number of hyperkalemic episodes requiring treatment with a diuretic during surgery and within 24 hours thereafter.

• Increase in plasma markers of inflammation (IL-6, MCP-1 and so on).

• Cell and differential counts in bronchoalveolar lavage fluid (BALF).

• Number and degree of activated alveolar macrophages (AMACs) in BALF.

• T-cells measurement and differentiation in BALF.

• Alveolar membrane thickness and surface area by open lung biopsy before and after CPB to indicate intracellular fluid accumulation.

• Degree of uncoupling the oxidative phosphorylation in musculus transversus abdominis and pulmonary tissue mitochondria.

• Number of tracheotomies 90 days after randomization.

• Hospitalization stay after surgery (days).

• Number of readmissions with a ventilation-related problem within 90 days after randomization.

• Reduction in PFT 90 days after randomization.

• 30- and 90-day mortality.

### Anesthesia, CPB, and lung protection

Anesthesia is induced with fentanyl (10 μg/kg), propofol (1 to 2 mg/kg) and cisatracurium (0.1 mg/kg) and maintained with sevoflurane (0.5% to 3%) and continuous infusion of remifentanil (15 to 30 μg/kg/hour). One peripheral vein, one radial artery and the internal jugular vein are cannulated. The ventilation mode is volume controlled with positive end-expiratory pressure (PEEP) set at 5 mmHg and lung recruitment is performed after CPB.

After heparinization (350 IU/kg, ACT >480 sec), normothermic CPB (36.5 to 37.0°C bladder temperature) is initiated as follows: in the ascending aorta an angled arterial cannula is placed (DLP 24 FR, Medtronic, Minneapolis, Minnesota, USA) and the right atrial appendage is provided with a two-stage venous cannula (36/46 FR, Medtronic). A membrane oxygenator (Capiox RX25, Terumo, Tokyo, Japan) and a roller pump (Stockert S5, Sorin Group, Milano, Italy) are used for perfusion with laminar flow. The arterial line includes a 40-μm filter (AL06, Pall, Port Washington, NY, USA). Pump flow is 2.4 L/minute/m^2^ body surface area. Mean arterial pressure is kept between 40 and 60 mm Hg.

In group I, the main pulmonary artery is provided with a straight arterial cannula (14 FR DLP, Medtronic), the aorta is cross-clamped, cardioplegia administered and pulmonary perfusion initiated at 300 to 400 ml/minute from the CPB circuit. We aim to maintain a pulmonary flow equaling about 10% of the patient’s total flow resulting in an overall flow rate of 110% until cross clamp release. The blood returning from the pulmonary circuit is drained to the venous reservoir via a left atrial vent (VT-53218, CalMed Laboratories, Costa Mesa, CA, USA). During CPB the lungs are not ventilated and that applies also to groups II and III.

The pulmonary perfusion pressure (PPP) is monitored online via the pulmonary cannula. We aim to maintain a PPP equal to the patient's baseline pulmonary artery mean pressure (PAMP) and not to exceed 20 mmHg.

In the event of lack of volume, the pulmonary perfusion is stopped and subsequently resumed when the needed volume is re-established.

Before aortic cross-clamp release the pulmonary perfusion is terminated and the pulmonary cannula removed.

In group II, pulmoplegia with 2 L 4°C custodiol HTK solution is performed after cardioplegia via a pulmonary artery cannula. The PPP is monitored similarly to group I and used to guide the infusion rate. The surgical procedure can continue during the pulmoplegia that takes about 8 to 10 minutes. The blood and solution returning from the pulmonary circuit to the left atrium is sucked and filtrated by a cell saver. The custodiol HTK solution is discarded and the remaining red blood cells are returned to the patient.

In group III, standard CPB is performed as described.

For groups I, II and III the residual blood in the CPB circuit is retransfused to patients after CPB. In case of excesses, fluid ultrafiltration (BC60 plus, Maquet, Wayne, NJ, USA) is performed during rewarming.

In group IV, TAVI is performed according to current guidelines at Rigshospitalet (ClinicalTrials.gov: NCT01057173).

### Blood samples

In all patient groups, blood samples for inflammatory indicators are collected from the pulmonary artery catheter: (1) after anesthesia induction; (2) at CPB start; (3) after declamping of the aorta; (4) 10 and 20 minutes after CPB; (5) at the end of surgery; (6) 120, 240, and 360 minutes after CPB; and (7) 24 hours post anesthesia induction. A total of 3 ml of blood is placed in ethylenediaminetetraacetate coated tubes, immediately centrifuged (or kept on ice for a maximum of 60 minutes) for 10 minutes at 1,800 rpm and cooled to 4°C. Plasma is extracted and stored in polypropylene test tubes at −80°C until assayed. Blood samples and measurements for determination of the different oxygenation values are obtained at time points 1, 4, 6 and 7.

Thoracic electrical impedance is measured at time points 1 through 7.

### Bronchoalveolar lavage

Two bronchoalveolar lavages (BAL) are performed using a flexible video 6 mm bronchoscope after induction of anesthesia and following termination of CPB [17]. Lidocaine gel (20 mg/g) is applied to the tip of the bronchoscope before insertion and the bronchoscope is wedged into a segment of the medial part of the right middle lobe. Sterile isotonic saline at 37°C is instilled in four aliquots of 60 ml, immediately aspirated (low suction <100 mmHg), and pooled into a sterile container on ice to obtain a BAL specimen.

Pooled non-filtered BALF is centrifuged in tubes containing a carrier protein (1% BSA in PBS) at 3,500 rpm at 4°C for 15 minutes and the supernatants are kept at −80°C until analysis. Total and differential cell counts are analyzed within 60 minutes from pooled non-filtered and non-centrifuged BALF.

### Oxygenation index and alveolar–arterial oxygen gradient

Blood samples for arterial gas analysis are processed immediately using a calibrated blood gas system (Radiometer, ABL 825, Broenshoej, Denmark). The oxygenation index is:

(1) $$\mathrm{OI}=\frac{F_{i}O_{2}\times \mathrm{MPAW}}{P_{a}O_{2}}$$

where F_i_O_2_ is the fractional concentration of oxygen in the inspired air and MPAW is the mean airway pressure.

We also calculate the pulmonary membrane diffusing capacity for oxygen (DmO_2_) as:

(2) $$D_{m}O_{2}=\frac{P_{A}O_{2}{-P}_{a}O_{2}}{\overset{\cdot }{Q}\left(C_{a}O_{2}{-C}_{v}O_{2}\right)}$$

where P_A_O_2_ is the partial pressure of alveolar oxygen, P_a_O_2_ the partial pressure of arterial oxygen, $\overset{\cdot }{Q}$ cardiac output, C_a_O_2_ the oxygen concentration of arterial blood from the left atrium and C_v_O_2_ the oxygen concentration of venous blood from the pulmonary artery.

### Lung and muscle biopsies

Two open lung biopsies will be obtained. One from lingula at the commencement of CPB and one from the right middle lobe before weaning from CPB. The lung tissue will be divided into three specimens: 1) will be fixed with formaldehyde for histological examination; 2) will be submerged immediately into liquid nitrogen and stored in polypropylene test tubes at −80°C until assayed by PCR; and 3) will be placed in relaxing medium (as described below for the muscle biopsy) until assayed for mitochondria function.

When the thorax is opened a biopsy will be obtained from the musculus transversus abdominis and placed in relaxing medium (10 mM Ca-EGTA buffer, 0.1 μM free calcium, 20 mM imidazole, 20 mM taurine, 50 mM K-MES, 0.5 mM DTT, 6.56 mM MgCl2, 5.77 mM ATP, 15 mM phosphocreatine, pH 7.1) at 2 to 4°C. Individual fiber bundles will be separated with forceps and then permeabilized for 30 minutes in 3 ml of ice-cold relaxing medium with saponin (50 μg·ml^-1^). Muscle bundles will be measured for wet weight and placed into the respirometer (Oxygraph-2 k, Oroboros Instruments, Innsbruck, Austria) containing a medium including EGTA (0.5 mM), MgCl_2_·6H_2_O (3 mM), K-lactobionate (60 mM), taurine (20 mM), KH_2_P0_4_ (10 mM), HEPES (20 mM), sucrose (110 mM), BSA (1 g/l) at a pH of 7.1 and assayed for mitochondria function.

### Criteria for extubation and discharge from the ICU

The oral tracheal intubation time is defined as from the end of surgery until extubation in the ICU in whole hours. The patient is extubated when the following criteria are met:

Pressure support ventilation (PSV) with support ≤8 cmH_2_O

FiO_2_ ≤0.40

SpO_2_/SaO_2_ ≥0.94%

pH ≥7.34

PEEP ≤5 cm H_2_O

Ventilation rate 10 to 12 L/minute

Bleeding from chest drains ≤200 ml/time

Blader temperature ≥36°C

The patient is awake and alert, pain free, hemodynamically stable with sufficient cough reflexes and swallowing movements.

On the following morning the patient is discharged from the ICU to the surgical ward if there is:

Uncomplicated anesthesia and recovery process in the ICU.

Blood loss ≤500 ml in patients with a preoperatively normal Hgb.

### Blinding

Patients and personnel in intensive care, recovery room, and ward are blinded to which group the patient is randomized. The experimental setup, however, does not allow blinding of the surgeons, anesthesiologists, and other staff members within the operating room. It will be emphasized that the involved staff does not report or disclose the randomization, for example, it will not be entered into the patient’s file.

Blood samples, BALF, and lung biopsies will be labeled with a unique patient number (starting 101) and personnel carrying out analyses will be blinded to which group the patient is randomized. Several of the other outcome variables cannot, however, be blinded but represent objective measures established at a fixed time. Decision makers on when to extubate a patient or whether FiO_2_ and other ventilator settings should be changed will be blinded to the allocation of the patient.

The patient's case report form (CRF) will be completed by the project coordinator or other personnel in accordance with the trial master file (TMF) list of tasks. The CTU will conduct blinded, double entry of the CRF data and blinded statistical analyses in which the intervention groups are coded as A, B, C, and D will be performed. Based on this analysis two blinded conclusions will be established: firstly assuming that groups A + B represent the intervention groups will be compared with data from groups C + D representing the control groups; and secondly the reverse scenario is assumed. Hereafter, and only then, will the groups be unmasked.

### Serious adverse reactions

Serious adverse reactions (SARs) described with the use of the custodiol HTK solution include electrolyte imbalance (hyponatremia, hypocalcemia, hyperkalemia, hypermagnesemia) and fluid overload [13]. Such manifestations can also be related to CPB but if they manifest, they will be recorded in the CRF and after an independent statistician has evaluated the data reported to the Ethics Committee and Danish Health & Medicines Authority. Furthermore, such events will be compared among the patients included in the two active trial groups. Yet, we consider that the amount of infused custodiol HTK solution is small (maximum 100 ml). Under all circumstances the Sponsor will provide a yearly report to the Ethics Committee and the Danish Health & Medicines Authority.

Suspected unexpected serious adverse reactions (SUSARs) will be defined as SARs not described in the summaries of product characteristics for custodiol HTK solution [13]. SUSARs will be reported by the trial site investigator to the Sponsor within 24 hours. The Sponsor ensures that all information on SUSARs that are fatal or life-threatening will be recorded and reported to the Danish Health & Medicines Authority and Dr. Franz Köhler Chemie GmbH as soon as possible and no later than within seven days after the sponsor becomes aware of such side effects.

All other SUSARs will be reported to the Danish Health & Medicines Authority within 15 days after the Sponsor is informed of such events. When reporting SUSARs the e-form on the Danish Health & Medicines Authority’s website is used.

Patients in the pulmonary perfusion group will also be followed for 24 hours during which, for example, bleeding, the number of blood transfusions, and the need for reoperation due to hemorrhage is recorded.

For patients undergoing cardiac surgery, the total time in the operating theatre is locally 6 to 8 hours, the time requiring mechanical ventilation is 10 to 12 hours, and the patients spend 7 to 10 days in hospital. All such data will be recorded in the CRF and reported to the Ethics Committee and Danish Health & Medicines Authority if it deviates markedly.

Events directly related to the surgery and events directly related to the normal course for this type of patient will be recorded but not reported as incidents. These events are: apoplexia cerebri, transient cerebral ischemia, acute myocardial infarction, heart failure, pericardial tamponade, pericardial effusion, arrhythmias, deep vein thrombosis, pneumothorax, emphysema, pleural effusion, pneumonia, subcutaneous emphysema, mediastinitis, anemia, pain, nausea, generalized weakness, constipation, esophagitis, ileus, gastric ulcer, acute liver damage, acute tubulointerstitial nephropathy, sepsis, wound infection and others.

### Safety

Discontinuation of the trial for an individual patient: Patients will be withdrawn from the interventions in the PP-Trial protocol if SARs or SUSARs occur.

Discontinuation of the whole trial: For a SUSAR or in case there is raised suspicion that a SAR may be related to the experimental treatment, this will be reported to the Ethics Committee and the Danish Health & Medicines Authority. There will, however, not be any interim analyses during the trial and the trial will not be terminated before the estimated sample size has been reached due to apparently beneficial effects of the interventions.

Yet, the trial will be terminated if results from other trials within the period of the trial show clear benefit or harm with one or more of the interventions.

### Patients’ withdrawal

Patients who are withdrawn from the PP-Trial trial protocol (see Safety) will be followed and analyzed the same as the remaining patients. Patients may withdraw their informed consent to participate in the trial at any time. The patients who withdraw their consent to participate in the trial will be asked for permission to obtain data for the primary outcome measure. If the patient declines, however, no data will be collected. All randomized patients will be reported, and all data available with consent will be used in the analyses. If appropriate, multiple imputation will be used [18]. If there are patients with missing data for the primary outcome measure, new patients will be randomized to obtain the full sample size.

Patients who are transferred to another ICU will be followed up for the primary outcome measure and all other assessable secondary outcome measures.

### Sample size estimation

Previously, a randomized clinical trial of pulmonary perfusion with oxygenated blood versus standard CPB [11] and a randomized clinical trial with custodiol HTK solution pulmoplegia versus standard CPB [12] has been performed. The primary outcome measure for these trials was the PF ratio defined as PF = P_a_O_2_/F_i_O_2_.

For the trial of pulmonary perfusion with oxygenated blood versus standard CPB the standard deviation (SD) for the PF ratio was 50 mmHg and the mean difference was 55 mmHg. For the trial of custodiol HTK solution pulmoplegia versus standard CPB, the SD for the PF ratio was 110 mmHg and the mean difference was 150 mmHg.

This experiment has theoretically six possible comparisons with randomization to three groups and a secondary control group represented by TAVI. However, for practical reasons five comparisons will be performed as this trial, based on the sample size estimation, does not include enough patients to perform a comparison between pulmonary perfusion with oxygenated blood and custodiol HTK solution. With multiple comparisons the risk of a type 1 error increases and to limit the family wise error rate to 0.05, we set the significance level to α = 0.01 for each of the five comparisons planned. The power 1 - β (β being the risk of a type 2 error set to 0.20) of each comparison is set to 80%.

Using the Dupont & Plummer power and sample size calculator [19], the required number of patients to detect or reject a relevant mean difference of 50 mmHg between pulmonary perfusion with oxygenated blood versus standard CPB is estimated at 30 patients for each group. The required number of patients to detect or reject a relevant mean difference of 150 mmHg in the PF ratio during the postoperative period between pulmonary perfusion with custodiol HTK solution versus standard CPB is estimated at 14 patients for each group. We have therefore chosen to set N = 4 × 30 = 120.

If the PF ratios in the above two interventions both prove to be superior to standard CPB, it is to be expected that the difference in the PF ratio between pulmonary perfusion with oxygenated blood or custodiol HTK solution will be less than the differences between the PF ratio of the two interventions and the PF ratio of the standard CPB. With a fixed N = 120, pulmonary perfusion with oxygenated blood versus custodiol HTK solution pulmoplegia yields a power of 67% to detect or reject a mean difference of 55 mmHg between the two interventions and, therefore, needs to be assessed there from. To obtain a power of 80% for the detection or rejection of a mean difference of 55 mmHg in comparison with pulmonary perfusion with oxygenated blood or custodiol HTK solution, with a risk of type 1 error of 1%, would require 52 patients in each group.

### Statistical analysis plan

The differences between the groups according to the primary outcome measure of OI, as mentioned measured six times during the postoperative period, will be tested with a mixed-effects model. For other continuous outcomes we will use analysis of variance (ANOVA). If the ANOVA is significant, a Student's *t*-test for unpaired groups will be used to locate deviating values. For the mixed-effects model and the variance analyses *P*-values less than 0.05 are considered to be statistically significant. For the Student’s *t*-test, *P*-values less than 0.01 are considered to be statistically significant. The difference between groups of the frequency of patients with binary outcomes including frequency of patients with one or more adverse events will be tested with a Chi^2^ test.

Trial results will be analyzed according to the modified intention to treat principle [17]. Post-randomization exclusion will be allowed only for patients not undergoing CPB dependent heart surgery. Further, per-protocol analyses will be performed excluding patients with major protocol violations defined as: 1) patients who were randomized to an intervention but did not receive any intervention; 2) patients who received an incorrect intervention; or 3) patients randomized to standard CPB who received preoperative pulmonary perfusion; and 4) patients who violated one or more inclusion or exclusion criteria.

With data missing on more than 5% of the patients (12 patients) a complete case analysis will be made, provided that the missing data can be assumed to be at random and that Little’s test is not significant. If missing data cannot be assumed to be at random and Little’s test is significant, multiple imputations will be performed assuming missing data at random [20].

### Data registration

Data will be registered in the CRF from patient notes (source) by trial or clinical personnel under the supervision of the trial investigators. From the CRFs the trial database (CTU) will be established by double data entry to minimize errors when converting data from paper to an electronic database. The following data will be registered:

Pre-operation characteristics (all obtained from hospital notes):

• National identification number, sex, ethnicity, age at randomization, height in cm, weight in kg, co-morbidities (previous cardiac or lung surgery, previous thoracic irradiation, previous admission for heart failure, myocardial infarction or stroke, asthma, COPD Y/N, chronic treatment for arterial hypertension, hypercholesterolemia or diabetes Y/N, cancer Y/N, and habitual p-creatinine and estimated glomerular filtration rate (eGFR)).

• A detailed heart and lung status including results from coronary angiography, echocardiography and PFT.

• Results from pre-operation blood samples (standard laboratory values), alcohol and medication status.

Per-operative data:

• Heart rate, mean arterial blood pressure, PAMP, left arterial pressure, central venous pressure, PPP during perfusion with oxygenated blood or custodiol HTK solution, cardiac rhythm, central venous oxygen saturation, cardiac output, PEEP, mean airway pressure, fraction of inspired oxygen and so on.

• Operating, perfusion and aortic cross clamp time, thoracic impedance, end surgical fluid status, arterial and mixed venous blood gases post induction, at start of CPB, at aortic cross-clamp release, and post CPB until 24 hours post anesthesia induction.

Post-operative data:

• Cardiac status (arrhythmias, myocardial infarction, persisted pacemaker need, direct current conversion, treated pericardial effusion, maximum levels of creatine kinase muscle and brain (CKMB) and troponin T (TnT), coronary angiography and/or angioplasty), neurologic status (stroke, convulsions, comatose >24 hours), respiratory status (total intubation time, reintubation, ventilator associated pneumonia, pneumothorax, pneumonia, treated pleural effusion) with blood gases two, four and six hours post CPB and the next morning after surgery, hemorrhage (amount 24 hours post operation in milliliters, amount in milliliters and type of blood transfusion), gastrointestinal status (ulcus, ileus, intestinal ischemia), renal status (renal replacement therapy, maximal creatinine).

• The total hospitalization time in days, ICU-LOS in hours, readmission to ICU, the need of and reason for reoperation, blood samples (standard laboratory values) and medication status on discharge from the Department of Cardiothoracic Surgery and to where the patient is discharged (home, local hospital or an organ specific department).

90 days after randomization data:

• Cardiac status (New York heart association (NYHA) and canadian cardiovascular society (CSS) classification, arrhythmias, myocardial infarction, persistent pacemaker need, heart failure, peripheral ischemia, venous thrombosis), sternal problems (loose, pain, resuturing), respiratory status (PFT, admission to hospital due to cough, shortness of breath and/or sputum, exacerbation of COPD, pneumonia), medication status.

• Total days of dialysis-dependency (any form of filtration or hemodialysis) summarized at day 90 from hospital notes or registries.

• Dialysis-dependency at day 90 defined as need of renal replacement therapy after day 90 post-randomization.

• Survival status obtained from hospital or civil registries.

• If the patient is deceased, date of death.

During the entire PP-Trial:

• SARs (Y/N for every day) including severe bleeding or serious allergic reactions defined as urticaria associated with worsened circulation (20% decrease in blood pressure or 20% increase in vasopressor dose), increased airway resistance (20% increase in the peak pressure on the ventilation, clinical stridor or bronchospasm).

1/2 and 1 year after randomization:

• Survival status obtained from hospital or civil registries.

### Data handling and retention

Data will be handled according to the Danish Data Protection Agency. All original records (including consent forms, CRFs, SUSAR reports and relevant correspondence) will be retained at the trial site or CTU for 15 years to allow inspection by the good clinical p ractice (GCP) unit at the Copenhagen University or local authorities. The trial database will be anonymized if requested by the authorities.

### Monitoring

The trial will be externally monitored (the GCP unit at University of Copenhagen) to GCP standards according to the EU directive 2001/20. Trial site investigators will provide access to source data according to the Clinical Trial Agreement.

### Ethics

The trial will adhere to the Helsinki Declaration II and the national laws of Denmark. The PP-Trial is approved by the Committees on Biomedical Research Ethics of The Capital Region of Denmark protocol no. H-1-2012-024, the Danish Medicines Agency journal no. 2012024017 (EudraCT no. 2011-006290-25, protocol code 4141), the Danish Data Protection Agency journal no. 2011-41-7051 and registered at ClinicalTrials.gov NCT01614951.

Patients will be enrolled only after informed consent.

No biological material will be collected for the trial, thus no bio-bank will be formed.

### Data analyses and publications

An independent statistician will perform the data analysis prior to breaking the randomization code. Based on these masked analyses of the primary and secondary outcome measures two abstracts with conclusions will be written by Katrine Bredahl Buggeskov. The randomization code will then be opened and a final manuscript written including the correct of the two pre-made abstracts. The manuscript will be submitted (to one of the major clinical journals) regardless of the results and the trials reported according to the Consolidated Standards of Reporting Trials (CONSORT) statement from 2010 [21]. Authorship will be granted depending on personal input according to the international committee of medical journal editors (ICMJE) definitions. All investigators will be acknowledged. Funding sources will have no influence on data handling, analysis or writing of the manuscript. Sib studies will be allowed if approved by the steering committee.

### Timeline

2010 to 2011: Applications for funding, ethical committees and medicines agencies, development of CRF and data management tools and development of monitoring plan.

2012 to 2014: Inclusion of patients.

Early 2015: Data analyses, writing and submission of the main manuscript for publication.

### Collaborators

The CTU has developed the internet based randomization system and the trial database together with the steering committee.

The GCP unit at University of Copenhagen has developed the monitoring plan and coordinated monitoring.

### Finances

The trial is funded by the Lundbeck Foundation, NordMedica, Aase & Ejnar Danielsens Fond and resources at trial sites. The funding sources have no influence on the trial design or data collection, management, analysis or reporting. The patients are covered by local insurance at the trial site.

### Perspective

The project combines interdisciplinary expertise in cardiovascular surgery, cardiothoracic anesthesiology, and cell and molecular biology. The involved patients are a high-risk cardiac surgical group because of their preoperative reduced lung function. There are currently no methods to mitigate the deterioration of lung function after cardiac surgery in this patient group. In the PP-Trial we use sophisticated intensive care and innovative monitoring in the form of invasive and non-invasive monitoring, clinical physiological examinations and biochemical, cell biological, immune-histochemical and histological analyses.

It is expected that trial results can contribute in innovative ways to: 1) a better understanding of pulmonary physiology, hemodynamics, and the role of lung inflammation during cardiac surgery; 2) a randomized clinical trial by intervention with pulmonary perfusion with oxygenated blood or custodiol HTK solution to improve the postoperative oxygenation ability for cardiac surgical patients with COPD; 3) develop a template for classification of cardiac surgical patients as low or high immunologic responders, making it possible to individualize patient treatment with a focus on reducing the CPB-triggered inflammatory response deleterious effects on the organs; and 4) to determine if the respiratory failure in TAVI patients is mainly due to an inflammatory response or to pump failure.

The results may be used as a precursor to a larger randomized multicenter trial and in other patient groups with, for example,a major surgical trauma, expected long CPB-time (extracorporeal membrane oxygenation treatment), and in severe sepsis/sepsis shock where our lung protective modalities may be able to reduce the pulmonary driven part of the immunologic response, and thereby diminish the degree of pulmonary failure and reduce mortality.

## Discussion

The PP-Trial will provide important data on the use of pulmonary perfusion with oxygenated blood or custodiol HTK solution in patients with COPD undergoing cardiac surgery. An alternative strategy could be cooling of relevant organs leading to an overall reduced cell turnover [5].

The PP-Trial has its limitations due to its inherent exploratory nature with the primary outcome of OI being a surrogate outcome. However, it is important to address the fact that patients with COPD, and especially patients with moderate to severe COPD, may be functionally incapacitated by hypoxemia, SIRS and prolonged recovery, and even an increased number of postoperative complications and death may be reflected by the degree of hypoxemia and SIRS. The sample size does not aim for confirmatory conclusions on the effect on important patient outcomes, such as mortality, cardiovascular complications, and risk of pneumonia and sepsis, which need a large trial with a low risk of bias, but the PP-Trial is considered an important feasibility trial paving the road for a possible pragmatic multicenter confirmatory trial.

## Trial status

The trial was registered 6 June 2012. Patients are enrolled from the Department of Cardiothoracic Surgery at Rigshospitalet, University of Copenhagen, Denmark. We expect to include two patients per week (holidays excluded) and to finish inclusion in 1 ½ years.

Presently (1 February 2013), 425 patients have been screened and 36 randomized.

## Abbreviations

AMAC: alveolar macrophages; ANOVA: analysis of variance; ARDS: adult respiratory distress syndrome; AV-difference: arterio-venous difference; AVR: aortic valve replacement; BAL: bronchoalveolar lavage; BALF: bronchoalveolar lavage fluid; BSA: bovine serum albumin; CABG: coronary artery bypass graft; CLD: chronic lung disease; COPD: chronic obstructive pulmonary disease; CPB: cardiopulmonary bypass; CRF: case report form; CTU: Copenhagen trial unit; DLCO: diffusion capacity for carbon monoxide; ECMO: extracorporeal membrane oxygenation; EF: ejection fraction; eGFR: estimated glomerular filtration rate; Euroscore: European system for cardiac operative risk evaluation; EVLW: extravascular lung water; FEV_1_: forced expiratory volume in 1 second; HLM: heart-lung machine; HTK: histidine-tryptophan-ketoglutarate; ICU-LOS: intensive care unit-length of stay; IL-6: interleukin 6; LAP: left atrial pressure; MAP: mean airway pressure; OI: oxygenation index; PAMP: pulmonary artery mean pressure; PBS: phosphate-buffered saline; PCR: polymerase chain reaction; PEEP: positive end-expiratory pressure; PFT: pulmonary function test; PPP: pulmonary perfusion pressure; PP-Trial: Pulmonary Protection Trial; RES: reticuloendothelial system; SARs: serious adverse reactions; SD: standard deviation; SIRS: systemic inflammatory response syndrome; SUSAR: suspected unexpected serious adverse reaction; SWG: Swan-Ganz; TAVI: transcatheter aortic-valve implantation; TI: thoracic impedance; TMF: trial master file; VAP: ventilator associated pneumonia.

## Competing interests

The authors declare that they have no competing interests.

## Authors’ contributions

All authors made substantive contributions to the PP-Trial as trial site investigators and revised and gave final approval of the manuscript. KB drafted the manuscript. DS is the principal investigator and sponsor of the PP-Trial and designed the trial together with TJ, LW and KB who are also the members of the steering committee. JW and NH are members of the data management and safety committee.
